# Undiagnosed Pheochromocytoma Presenting as a Pancreatic Tumor: A Case Report

**DOI:** 10.1515/med-2020-0015

**Published:** 2020-02-20

**Authors:** Malgorzata Emilia Legocka, Sadegh Toutounchi, Ryszard Pogorzelski, Ewa Krajewska, Krzysztof Celejewski, Zbigniew Galazka

**Affiliations:** 1 Medical University of Warsaw, Warsaw, Poland; 2 Department of General, Endocrinological and Vascular Surgery, Medical University of Warsaw, Warsaw 02-097 Poland

**Keywords:** Pheochromocytoma, Hypertensive crisis, Abdominal tumor, Adrenal tumor, Intraoperative management, Laparoscopy, Adrenalectomy

## Abstract

Pheochromocytoma is a rare catecholamine-producing tumor of the adrenal gland. Patients with known pheochromocytoma undergoing surgery require preoperative treatment with alpha-blockers to reduce the risk of intraoperative complications related to catecholamine release. If undiagnosed, pheochromocytoma can lead to life-threatening surgical complications. We report the case of a patient with a suspected solid pseudopapillary neoplasm in the pancreatic tail, for whom pancreatoduodenectomy was scheduled. However, shortly after abdominal incision, hypertensive crisis developed and was followed by severe hypotension requiring intravenous vasopressors, which prompted discontinuation of the operation. Further diagnostic evaluation revealed marked elevations in urinary excretion of methylated catecholamines and suggested that the tumor was in fact a pheochromocytoma extending from the left adrenal gland. After preoperative treatment with doxazosin, the patient underwent lateral transperitoneal laparoscopic adrenalectomy, with no major complications and an uneventful postoperative course. The pathological report confirmed a diagnosis of pheochromocytoma. Due to the potential for life-threatening surgical complications in patients with pheochromocytoma not treated preoperatively with alpha-blockers, this tumor type should be included in the differential diagnosis of abdominal tumors of unknown origin.

## 1 Introduction

Pheochromocytoma is a catecholamine-producing tumor of the adrenal gland that occurs in less than 0.1% of the general population [1]. In clinical practice, pheochromocytoma is often overlooked, with less than half of patients with pheochromocytoma found on autopsy receiving that diagnosis during their lifetime [2]. Symptoms of pheochromocytoma can include hypertension, headaches, palpitations, pallor, and anxiety, which are caused by catecholamine release and often occur only episodically. In patients with pheochromocytoma, surgery can trigger life-threatening complications, such as hypertensive crisis and hemodynamic instability [3]. However, an exception to the classic presence of pheochromocytoma is ‘silent pheochromocytoma’, which does not exhibit classic symptoms. Abdominal CT scan also may not accurately delineate the organ site of the mass. Clinically, pheochromocytoma may mimic a neoplasm of the liver, kidney or pancreas [4, 5, 6]. Therefore, pheochromocytoma should be considered in retroperitoneal tumors of patients with nonspecific symptoms to promote the perioperative safety and administration of adequate treatment [7]. Technically, surgical resection of retroperitoneal tumors also has its risks due to aberrant vascularization. In those cases, surgeons should not underestimate the condition of the accessory arteries to prevent critical postoperative complications, such as liver necrosis [8,9]. We report the case of a patient with a hypertensive emergency during elective pancreatoduodenectomy that ultimately led to a diagnosis of pheochromocytoma.

## 2 Case report

**Figure 1:**
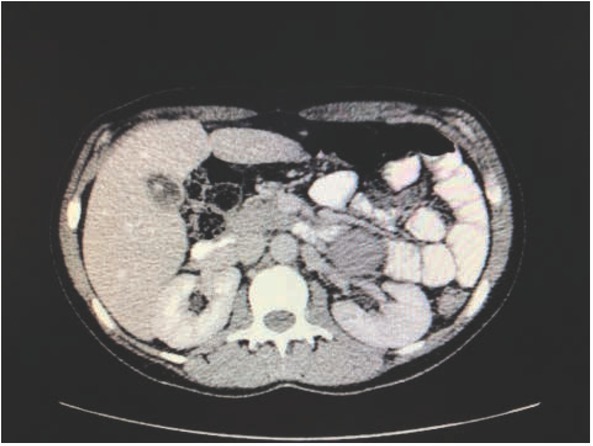
CT scan with suspected pancreatic tumor

An elective pancreatoduodenectomy was scheduled for a 35-year-old woman with a pancreatic mass suspected to be a solid pseudopapillary neoplasm (Fig. 1). Her medical history included two cesarean sections and moderately increased blood pressure (160/80 mm Hg), which was managed with ramipril. The blood pressure was 145/100 mm Hg on the day of surgery. During induction of anesthesia (with extradural injection of bupivacaine and epinephrine) and after application of mild pressure to the abdomen, the blood pressure increased suddenly to 266/167 mm Hg and, after abdominal incision, increased further to 288/180 mm Hg. Tissue preparation was stopped, and severe hypotension (blood pressure: 50/20 mm Hg) developed, which was managed with intravenous catecholamines. The operation was discontinued, and the patient recovered in the intensive care unit.

**Figure 2:**
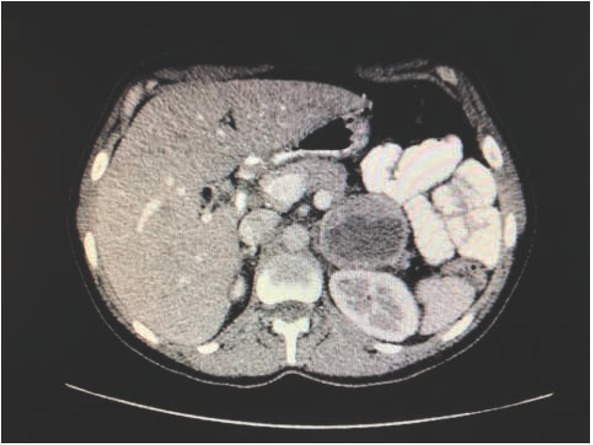
CT scan with tumor resembled pheo of left adrenal gland

Afterwards, the patient was referred to our department for further diagnostic evaluation. A detailed medical history revealed episodes of generalized weakness, shortness of breath, palpitations, and skin pallor, lasting approximately 2 minutes, which had occurred once or twice monthly during the previous year. Symptoms were worsened by sitting and improved by walking and deep breathing. Moreover, we learned that the patient had previously been hospitalized with increased blood pressure during pregnancy. The workup showed marked elevations in urinary excretion of methylated catecholamines (Table 1). Based on previously obtained abdominal computed tomography and magnetic resonance imaging, a team of radiologists, internists, and surgeons agreed that the pancreatic tumor resembled a pheochromocytoma extending from the left adrenal gland (Fig. 2). Thus, the patient was scheduled for lateral transperitoneal laparoscopic adrenalectomy.

**Table 1:** Urinary excretion of methylated catecholamines before and 6 months after adrenalectomy in a patient with pheochromocytoma

	Daily urinary excretion (μg/24 hours)		
	Normal range	Before adrenalectomy	After adrenalectomy
Normetanephrine	< 540	6226	197.2
Metanephrine	< 240	5934	97
3-methoxytyramine	< 426.4	696	174.9

**Figure 3:**
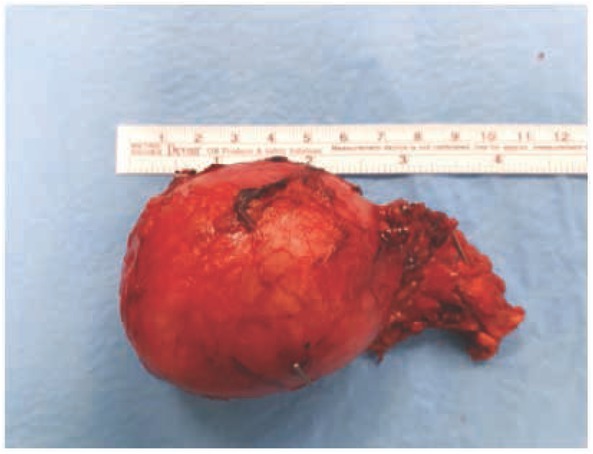
Excised tumor

**Figure 4:**
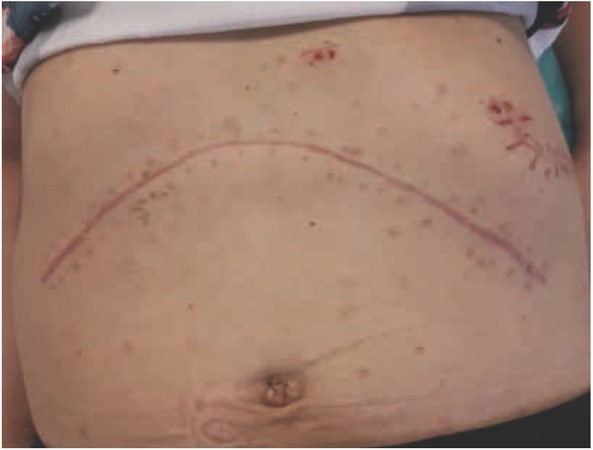
Incisions after 1st and 2nd surgery

The patient received preoperative doxazosin at a final dose of 4 mg four times daily for a 1-month period, which normalized blood pressure (110–120/70–80 mm Hg). Because the tumor was well vascularized, we used a harmonic scalpel for the adrenalectomy. Immediately prior to ligation of the adrenal vein, blood pressure increased to 310/120 mm Hg, which was managed with intravenous sodium nitroprusside. The operation lasted for 3 hours, and the excised tumor measured 5.7 cm × 4.5 cm × 5.0 cm (Fig. 3). After the operation, the patient was in good condition, with normal blood pressure, and was discharged after 5 days (Fig.4). Symptoms did not recur, and urinary excretion of methylated catecholamines was normal 6 months after the adrenalectomy (Table 1). Pathological examination revealed a pheochromocytoma that did not infiltrate the adrenal capsule or local blood vessels.

Ethical approval: The research related to human use has been complied with all the relevant national regulations, institutional policies and in accordance the tenets of the Helsinki Declaration, and has been approved by the authors' institutional review board or equivalent committee.

Informed consent has been obtained from patient included in this study.

## 3 Discussion

Elective surgery in patients with undiagnosed pheochromocytoma can be life-threatening [10]. Previous studies in this setting showed that hemodynamic instability occurs primarily during tumor manipulation and induction of anesthesia. Consistent with this observation, this patient’s blood pressure increased markedly after extradural anesthesia and manipulation of the abdomen. Other investigators reported severe hypotension after pheochromocytoma excision in patients not preoperatively treated with alpha-blockers [11]. In this case, severe hypotension developed during the first operation, when pheochromocytoma was not suspected, but not following tumor removal in the second surgery, after preoperative treatment with doxazosin. This finding underscores the importance of appropriate preoperative management in patients with suspected pheochromocytoma.

Surgery in patients with undiagnosed pheochromocytoma is associated with a high mortality rate of 8–40% [12]. In contrast, the mortality rate in patients with diagnosed pheochromocytoma who receive alpha-blockers preoperatively is much lower (0% in some series [13]). Thus, intraoperative suspicion of pheochromocytoma in a patient undergoing elective surgery should prompt dis-continuation of the operation.

Inappropriate management of hemodynamic instability in patients with pheochromocytoma may increase the risk of death. For example, use of selective beta-blockers to control tachycardia may cause acute heart failure with pulmonary edema, due to the vasoconstriction and resultant increase in blood pressure arising from the unopposed action of catecholamines on alpha-receptors [14]. Thus, alpha-blockers are a good choice for managing hypertensive crises if there is intraoperative suspicion of pheochromocytoma in patients not treated with such medication before surgery. However, in an analysis of published case reports, previously undiagnosed pheochromocytoma was suspected intraoperatively in only a quarter of patients, and all five patients who died had not received alpha-blockers during surgery [12]. In patients who receive alpha-blockers preoperatively, intraoperative blood pressure elevations should be managed with other intravenous antihypertensive drugs, such as sodium nitroprusside, as used in our hospital. Perioperative mortality is low in patients who undergo planned removal of pheochromocytoma, due to preoperative use of alpha-blockers to prevent hypertensive crises and intravenous fluids to reduce potential hypotension [15].

Laparoscopic adrenalectomy is as safe and effective for patients with pheochromocytoma as it is for patients with other adrenal tumors [16]. While laparoscopy is more technically challenging than open adrenalectomy, both techniques have a similar risk of complications [17].

## 4 Conclusion

Although pheochromocytoma is rare, it should be included in the differential diagnosis for patients with hemodynamic instability during surgery and in every case of retroperitoneal tumor. Surgical removal is the treatment of choice for pheochromocytoma, but patients should receive selective alpha-blockers for several weeks before the operation for perioperative safety. In cases where diagnosis of pheochromocytoma is not evident, it should be still considered to administer alpha-blockers before the surgery. Detailed medical history, adequate diagnostic workup, and experience of the management team are important for recognizing pheochromocytoma in patients presenting with abdominal tumors of unknown origin.
